# Role and mechanism of Integrin α5β1 in bone formation and disease

**DOI:** 10.3389/fcell.2025.1632710

**Published:** 2025-08-14

**Authors:** Xiaoying Li, Guangxuan Hu, Jinshi Guo, Bo Chang, Xuejie Yi, Tingting Yao

**Affiliations:** ^1^School of Sports Health, Shenyang Sport University, Shenyang, China; ^2^School of Physical Education, Liaoning Normal University, Dalian, China; ^3^School of Sports Science, Zhuhai College of Science and Technology, Zhuhai, China; ^4^Social Science Research Center, Shenyang Sport University, Shenyang, China

**Keywords:** integrin α5β1, bone formation, osteoporosis, osteoarthritis, bone metastasis

## Abstract

Integrin α5β1 is a key signaling protein between cells and the extracellular matrix. It plays crucial roles in biological processes such as cell adhesion, migration, and differentiation. Recent studies have shown that integrin α5β1 is significantly involved in bone formation and related diseases. Integrin α5β1 participates in the differentiation of mesenchymal stem cells into osteoblasts. It interacts with the CCN family and the bone morphogenetic protein pathway to upregulate the expression of osteogenic markers, promoting the formation of mineralization nodules. Additionally, it can mediate mechanical force stimulation to upregulate osteogenic gene expression and promote bone formation. In diseases such as osteoporosis, osteoarthritis, and bone metastasis, integrin α5β1 mediates abnormal cell-matrix adhesion and migration, promoting pathological bone resorption and inhibiting bone formation, thereby exacerbating bone loss. Therefore, integrin α5β1 may be a potential therapeutic target for these bone diseases. Elucidating its mechanism of action will help understand the homeostatic regulation of bone metabolism and provide ideas for the development of novel therapeutic strategies for skeletal diseases.

## 1 Introduction

The skeletal system is the body’s supporting framework, composed of compact bone and cancellous bone, and includes multiple cell types—osteocytes, osteoblasts, and osteoclasts—that together maintain bone growth, repair, and remodeling (Stegen and Carmeliet, 2024). Skeletal health relies on a precise balance between bone formation and bone resorption, a dynamic process regulated by a complex molecular network (Siddiqui and Partridge, 2016). When this balance is disrupted, it can lead to bone-related diseases such as osteoporosis and osteoarthritis, imposing a health burden on over 200 million people worldwide (Whittier et al., 2022; Glyn-Jones et al., 2015; Carey et al., 2022). Bone tissue is an organ highly sensitive to mechanical stimuli, and its metabolic activity is significantly influenced by biomechanical factors (Morgan et al., 2018). Studies have shown that bone cells, by sensing mechanical load, regulate both osteogenesis and osteoclastogenesis, but the underlying molecular mechanisms remain incompletely understood (Robling and Bonewald, 2020; Delgado-Calle and Bellido, 2022). In recent years, the integrin family—key mediators of cell–matrix adhesion—has attracted widespread attention for its role in the mechanical sensitivity of bone tissue (Zhao et al., 2022; Yang et al., 2024).

In the integrin family, α5β1 functions as the principal fibronectin receptor and exhibits distinctive roles in osteogenesis. Clinical studies have demonstrated that dysregulated α5β1 expression is closely associated with various bone disorders: its expression is downregulated in the bone tissue of osteoporosis patients, whereas it is upregulated in the chondrocytes of individuals with osteoarthritis (Marie, 2013; Sumsuzzman et al., 2022). Although the importance of integrin α5β1 in bone biology has become increasingly evident, a comprehensive understanding of its precise mechanisms of action, regulatory networks, and clinical application potential remains lacking. Notably, the rapid advancement of bone biology research techniques has yielded a plethora of novel findings on integrin α5β1 in recent years, underscoring the need for a systematic synthesis and analysis of these data (Sun et al., 2018; Shao et al., 2019; Riquelme et al., 2021; Mao et al., 2023).

This review aims to systematically elucidate the mechanistic and clinical significance of integrin α5β1 in bone formation and bone-related diseases by: (1) summarizing its expression profiles in bone tissue and delineating its structure–function relationships; (2) analyzing the mechanotransductive signaling pathways it mediates and their roles in osteogenic regulation; (3) exploring its pathophysiological contributions to major skeletal disorders; and (4) evaluating current and prospective interventional strategies targeting α5β1. By integrating the most recent research advances, this review intends to establish a novel theoretical foundation and offer fresh perspectives for bone-biology research and the treatment of skeletal diseases.

## 2 Overview of integrin α5β1

### 2.1 Structural characterization of Integrin α5β1

Integrin α5β1, one of the 24 members of the integrin heterodimer family, is currently the only known α5 integrin (Pacifici et al., 1992). The α5 subunit in the α family of integrins and the β1 subunit in the β family are combined by non-covalent bond interaction, which together constitute its complete biological function (Rocha et al., 2018; Kanchanawong and Calderwood, 2023).

**FIGURE 1:**
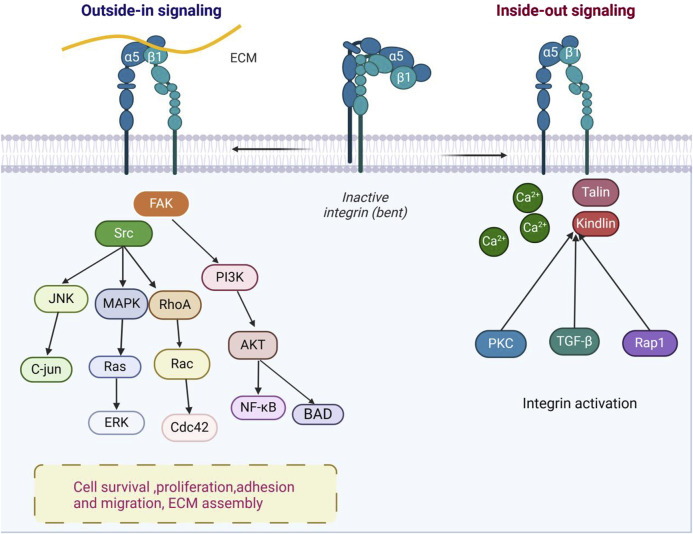
Integrin α5β1 bidirectional signaling pathway diagram. In the “outside-in” signaling pathway, integrin α5β1 binds to ligands such as ECM, activating downstream signaling pathways through molecules like FAK and Src, such as MAPK and PI3K. This regulates cell survival, proliferation, adhesion, and migration, as well as extracellular matrix assembly. In the “inside-out” signaling mechanism, integrin α5β1 activation promotes the regulation of interactions between cells and the ECM. Intracellular calcium levels, PKC, TGF-β signals, and key proteins such as Talin and Kindlin bind to the β1 tail of integrin, activating the conformational change of integrin α5β1 (figure generated through BioRender.com).

The integrin α5 gene (*ITGA5*) is indeed responsible for encoding the α5 subunit, which is located on chromosome 12q11. This subunit features specific domains, including the extracellular leg domain and the β-propeller domain. These domains are crucial for the function of integrin α5β1, particularly in cell adhesion and signal transduction (Luo et al., 2007). The α5 subunit can recognize the arginine-glycine-aspartic acid (RGD) motif in fibronectin (FN) and fibrinogen (Nagae et al., 2012). RGD sequences are common cell-adhesion signals in ECM proteins such as fibronectin (FN) (Corti and Curnis, 2011). Notably, the binding pocket of integrin α5β1 that recognizes the RGD motif is formed at the interface of the α5 and β1 subunits, rather than being located on either subunit alone. This structural arrangement enables α5β1 to specifically recognize and bind RGD-containing ECM proteins (Xia and Springer, 2014). The integrin β1 gene (*ITGB1*) includes a plexin-semaphorin-integrin (PSI) domain, a heterodimer domain, a βI domain with a metal ion-dependent adhesion site (MIDAS), and four epidermal growth factor (EGF)-like domains in its extracellular portion, and is located on chromosome 10p11.2 (Barczyk et al., 2010) (Figure 1). According to current research, the interaction between integrin α5β1 and its extracellular ligands relies on the MIDAS structure and divalent cations, with calcium ion (Ca^2+^) being an important cation for integrin α5β1 ligand binding (Carman and Springer, 2003).

### 2.2 Functional activities of Integrin α5β1

**FIGURE 2:**
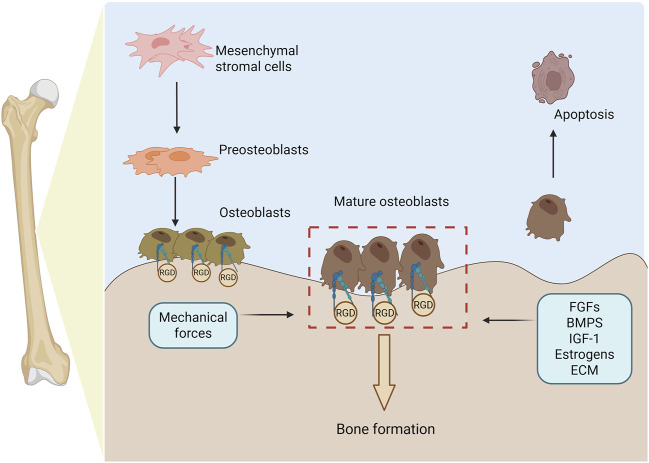
Osteogenesis is regulated by integrin α5β1. This process begins with mesenchymal stromal cells (MSCs) differentiating into pre-osteoblasts, which then mature into fully functional osteoblasts. Throughout this progression, α5β1 is indispensable for promoting osteoblast adhesion, lineage specification, and responsiveness to the extracellular matrix. Mature osteoblasts secrete bone matrix proteins to drive new bone formation and, upon completing their anabolic functions, undergo programmed apoptosis. Specifically, α5β1 binding to RGD-containing ECM ligands facilitates osteoblast adhesion and migration. Osteogenesis is further modulated by fibroblast growth factors, bone morphogenetic proteins, hormones, ECM composition, and mechanical cues—all of which converge on α5β1 regulation to fine-tune bone formation (figure generated through BioRender.com).

Integrin α5β1, as a key receptor on the cell surface, plays a crucial role in cell adhesion, migration, and signal transduction processes (Su et al., 2022). Its active state and ligand binding ability are regulated by various intracellular and extracellular factors, including changes in ion concentrations, modifications of intracellular signaling molecules (such as phosphorylation), and interactions with other cell surface or ECM proteins (Shattil and Newman, 2004; Legate and Fässler, 2009; Campbell and Humphries, 2011). These regulatory mechanisms determine the dynamic adaptability of integrin α5β1 in different cellular environments. In terms of signal transduction, α5β1 exhibits bidirectional signaling: it transmits ligand-derived cues from the ECM (e.g., fibronectin) into the cell *via* outside-in activation, inducing conformational changes and engaging downstream pathways such as FAK, Src, and PI3K; conversely, it undergoes inside-out activation when intracellular adaptors (e.g., talin or kindlin) bind its cytoplasmic tails, converting α5β1 to a high-affinity conformation and enabling reciprocal signal transmission (Kim et al., 2011; Shen et al., 2012) (Figure 1). Moreover, α5β1 interacts with diverse ligands—including SEMA7A, irisin, and EphA2—to regulate cytoskeletal dynamics, chemotaxis, and ECM remodeling (Liu et al., 2020; Spoerri et al., 2020; Bochicchio et al., 2021; Caliva et al., 2021; Finney et al., 2021; Myint et al., 2021; Hu et al., 2023). In bone tissue in particular, integrin α5β1 constitutes a critical determinant of osteogenesis (Figure 2).

## 3 Integrin α5β1 mediates bone formation

In bone biology, integrin α5β1, by virtue of its distinctive structure and signaling pathways, exerts specialized regulatory functions during bone development, remodeling, and repair (Campbell and Humphries, 2011; Myint et al., 2021; Su et al., 2022). Studies have shown that within the bone microenvironment, α5β1 interacts with ECM molecules such as fibronectin to not only provide mechanical support but also trigger a cascade of bone-specific signaling events that govern remodeling balance and mineralization (Shattil and Newman, 2004; Su et al., 2022). These roles are essential for maintaining skeletal health, and their disruption can give rise to various bone-related pathologies, including osteoporosis and osteoarthritis (Nagae et al., 2012; Rocha et al., 2018).

### 3.1 Integrin α5β1 mediates MSC differentiation

#### 3.1.1 The crucial role of Integrin α5β1 in MSC osteogenic differentiation

During osteogenesis, mesenchymal stem cells (MSCs) serve as the primary progenitors for bone formation. Integrin α5β1 mediates MSC differentiation into osteoblasts (OBs), as well as their migration and adhesion to bone surfaces, thereby maintaining bone homeostasis (Su et al., 2018). Osteogenic differentiation of MSCs is characterized by upregulation of key osteogenic transcription factors (e.g., Runx2) and osteoblastic markers—including alkaline phosphatase (ALP), osteocalcin (OCN), and type I collagen (Col1a1)—and culminates in calcium deposition within the ECM (Karsenty and Wagner, 2002; Lian et al., 2004).

Simvastatin (SVS), a member of the statin family, enhances expression of bone morphogenetic protein-2 (BMP-2) and thereby induces MSC osteogenesis (Chen et al., 2010). In BALB/c-derived bone marrow MSCs (D1 cells), siRNA-mediated knockdown of the α5 integrin subunit markedly attenuated SVS-induced osteogenic marker gene expression (BMP-2, Runx2, and OCN mRNAs), ALP activity, and calcium deposition, compared with cells transfected with a non-specific control oligonucleotide under SVS treatment (Shao et al., 2019). These findings demonstrate that integrin α5 is essential for SVS-promoted osteogenic differentiation.

CCN family proteins, as secreted extracellular matrix constituents, play critical roles in bone development and remodeling. Members of this family—such as CCN3 and WISP-1—promote MSC osteogenic differentiation primarily by enhancing BMP signaling pathways (Ono et al., 2011; Tan et al., 2012). BMPs, as pivotal members of the TGF-β superfamily, serve as core growth factors regulating bone formation and remodeling (Wu et al., 2024). Integrin α5β1 functions as a functional receptor for multiple CCN family members, mediating their pro-osteogenic effects (Ahmed et al., 2021). Studies have shown that CCN3 (also known as NOV) binds integrin α5β1 to activate phosphorylation of FAK and Akt, thereby upregulating the expression of key osteogenic transcription factors Runx2 and Osterix (Chen et al., 2019). Furthermore, CCN3 augments matrix mineralization by upregulating BMP-4 expression in osteoblasts (Urist, 1965; Chen and Lau, 2009). Tan et al. demonstrated that pre-treatment of MC3T3-E1 osteoblastic cells with an anti-α5β1 monoclonal antibody significantly inhibited CCN3-induced BMP-4 expression and nodule formation, confirming the necessity of α5β1 in CCN3’s osteogenic activity (Tan et al., 2012). WISP-1 (Wnt-induced secreted protein 1), another CCN family member highly expressed in bone tissue, contributes to skeletal formation and homeostasis (French et al., 2004). Ono et al. provided multiple lines of evidence for a specific, dose-dependent direct interaction between WISP-1 and integrin α5β1. Crucially, this interaction significantly enhanced BMP-2 binding to bone marrow MSCs (BMSCs). In human BMSCs overexpressing WISP-1, blocking α5β1 with a specific antibody markedly reduced BMP-2 binding and ALP expression, whereas an IgG control antibody had no effect (Ono et al., 2011). These findings indicate that WISP-1 potentiates BMP-2–BMSC interactions *via* integrin α5β1, although additional regulatory factors may modulate this complex.

Furthermore, from a genetic-engineering perspective, rat BMSCs overexpressing integrin α5β1 exhibited increased cell viability alongside decreased caspase-3 activity, indicating that upregulation of α5β1 not only enhances the osteogenic capacity of BMSCs but also improves their survival (Chen et al., 2018).

#### 3.1.2 Regulatory role of Integrin α5β1 in MSC multilineage differentiation

MSCs are multipotent progenitors capable of differentiating not only into osteoblasts but, under appropriate conditions, also into chondrocytes, adipocytes, and other lineages (Uccelli et al., 2008). As a key mediator of cell–ECM adhesion, integrin α5β1 plays a pivotal role in directing these diverse differentiation trajectories of MSCs.

During adipogenic differentiation, integrin α5β1 expression progressively declines, and its inhibition facilitates MSC commitment to adipocytes; conversely, α5β1 overexpression preserves an undifferentiated state and suppresses adipogenesis (Liu et al., 2005; Uetaki et al., 2022).In chondrogenic differentiation, α5β1 binds fibronectin fragments to regulate the adhesion and migration of chondrogenic progenitor cells, thereby influencing chondrocyte phenotypic stability (Loeser, 2014). In adipose-derived stem cells (ADSCs), the circular RNA circRNA-VGLL3 functions as a sponge for miR-326-5p, thereby relieving its repression of *ITGA5* and consequently enhancing osteogenic differentiation and new bone formation (Zhang et al., 2021). Moreover, ANGPTL2 is also expressed in chondrocytes—particularly within the resting and proliferative zones—and accumulates in the extracellular matrix (Kadomatsu et al., 2014). Mechanistically, ANGPTL2 exerts its effects by specifically binding the integrin α5β1 receptor, thereby activating the downstream p38 MAPK signaling pathway, which promotes chondrocyte differentiation and endochondral ossification. Experiments in Angptl2-knockout mice demonstrated that loss of ANGPTL2 delays long-bone growth from the neonatal to adult stages, indicating that the ANGPTL2–α5β1–p38 MAPK signaling axis plays a critical regulatory role in skeletal development (Tanoue et al., 2018).

The stromal vascular fraction (SVF) of human adipose tissue contains multipotent mesenchymal progenitors—adipose-derived stem cells (ASCs)—that give rise to osteogenic grafts with intrinsic angiogenic properties. In ASCs, the expression level of integrin α5β1 closely correlates with their osteogenic potential. α5β1-mediated ECM signaling preserves ASC progenitor characteristics, leading to a substantial increase in ERK1/2 phosphorylation and enhanced bone formation capacity (Di Maggio et al., 2017). Moreover, although MSCs also possess myogenic differentiation potential, the specific role of integrin α5β1 in this lineage commitment is poorly characterized in the current literature, representing a promising avenue for future research.

### 3.2 Integrin α5β1 mediates osteogenic differentiation

In vertebrates, intramembranous osteogenesis is essential for skeletal development and remodeling (Ko and Sumner, 2021). In the initial stages of intramembranous ossification, MSCs and pre-osteoblasts migrate from peripheral areas to the site of future bone formation, differentiate into OBs, and secrete bone extracellular matrix, which eventually mineralizes to form new bone (Dirckx et al., 2013; Thiel et al., 2018). Chemotaxis is the directed migration of cells along a gradient of extracellular molecules, known as chemotactic attractants (Majumdar et al., 2014). This process plays a key role in bone development and homeostasis maintenance (Devreotes and Janetopoulos, 2003).

Connective tissue growth factor (CTGF, also known as CCN2) is considered a genetic factor involved in endochondral ossification and exhibits chemotactic properties toward osteoblast lineage cells (Ono et al., 2008; Takigawa, 2013). Integrins are believed to be functional receptors for CTGF, with CTGF activating cell migration and adhesion through binding to integrin α5β1 (Kiwanuka et al., 2013). In experiments involving tension-induced parietal bone formation, integrin α5β1 expression was observed in both non-stressed osteoblasts and cells clustered at the leading edge of stressed osteoblasts. Notably, CTGF was detected not only in the extracellular matrix adjacent to *ITGA5*-positive cells but also within these cells. Further experiments demonstrated that *ITGA5* regulates the chemotactic effect of CTGF on MC3T3-E1 cells, and using neutralizing antibodies against *ITGA5* effectively inhibited CTGF-induced directional cell migration. These findings highlight the regulatory role of *ITGA5* in the osteogenesis process, particularly in CTGF-mediated cell chemotaxis and bone formation (Jiang et al., 2021).

Epidermal growth factor-like repeats and discoidin I-like domains 3 (Edil3) is an extracellular matrix protein that contains an RGD motif and is a ligand for integrins αvβ3 and αvβ5 (Feng et al., 2014). Previous studies found that inhibiting Edil3 gene expression resulted in craniofacial abnormalities in embryonic mice (Savontaus et al., 2004). The interaction between integrins and the ECM further promotes the formation of specific early genes in OBs. Among these interactions, the role of integrin α5β1 with fibronectin (FN) is crucial for pre-OBs to attach to the ECM and subsequently differentiate into mature OBs (Marie, 2013). Oh et al. assessed the expression of various integrins in MC3T3-E1 cells under standard growth medium conditions, finding that α5β1 was more prominently expressed compared to αvβ3 and αvβ5. Using blocking antibodies against integrin α5β1 revealed that Edil3-induced mineralization and OB-specific gene expression (ALP and OCN) were inhibited. To explore the potential mechanism of Edil3-induced osteogenic differentiation, MC3T3-E1 pre-osteoblasts were cultured in growth medium containing Edil3, and samples were collected at specific time points (at 0.five to one h and 24 h) for analysis. Preliminary results showed that the phosphorylation levels of Akt, ERK, and p38 peaked within 0.5–1 h, significantly higher than in untreated controls. Additionally, the expression of Runx2 protein significantly increased 24 h after the Edil3 treatment. Subsequently, to investigate how Edil3 influences Runx2 expression through the ERK pathway, Edil3 was added to the growth medium in the experiment. The results showed a time-dependent increase in Runx2 protein expression (Oh et al., 2017). Therefore, Edil3 not only regulates osteogenesis through its interaction with αv-class integrins, but may also activate Runx2 *via* integrin α5β1-mediated signaling pathways (such as ERK), thereby promoting osteogenic differentiation and highlighting its synergistic, multi-pathway role in bone formation.

The above studies indicate that integrin α5β1 plays a crucial regulatory role in the differentiation of osteoblasts. It not only participates in cell chemotaxis and migration but also acts as a molecular bridge mediating the activation of osteogenic signaling pathways, such as ERK/Runx2, by ECM proteins (such as CTGF and Edil3). Additionally, it can sense and respond to external physical environment signals (such as ECM stiffness) to promote the differentiation of MSCs into osteoblasts. Blocking its function can inhibit this process. Integrin α5β1 plays an indispensable role in osteogenic differentiation by integrating molecular, cellular, and environmental regulation.

### 3.3 Integrin α5β1 mediates mechanotransduction for bone formation

Bone formation is dependent on mechanical stimulation. In the absence of mechanical stimulation, bone structure significantly weakens, leading to disuse osteoporosis and increased fracture risk (Wang et al., 2022). In the bone’s lacunocanalicular network, osteocytes, osteoblasts (OBs), and mesenchymal stem cells (MSCs) serve as the primary sensors of mechanical and physical stimuli (Riquelme et al., 2020; Chang et al., 2022). Through a variety of mechanosensitive molecules—most notably integrin family proteins—these cells detect and transduce mechanical signals to regulate bone metabolic homeostasis (Kanchanawong and Calderwood, 2023).

Different modes of mechanical stimulation exert distinct effects on bone cells. Pulsatile fluid flow shear stress (periodic oscillatory flow) markedly alters the cytoskeletal architecture, nuclear morphology, and volume of murine calvarial osteoblasts, concomitantly upregulating α5 integrin at both the mRNA and protein levels (Jin et al., 2020). In contrast, steady fluid flow shear stress (FFSS) activates Runx2 *via* enhanced ERK1/2 phosphorylation, thereby increasing the expression of osteogenic markers. Notably, blockade of β1 integrin–ECM engagement inhibits FFSS-induced ERK1/2 and FAK activation (Liu et al., 2014), underscoring the critical role of α5β1 in mechanotransductive signaling.

In the molecular framework of mechanotransduction in bone cells, integrin α5β1 serves as a mechanosensor: upon sensing fluid shear stress, it activates the PI3K/AKT signaling cascade, which in turn regulates connexin-43 (Cx43) hemichannel function (Riquelme et al., 2021). Cx43 hemichannels are mechanoresponsive conduits that allow the exchange of signaling molecules <1.2 kDa—such as prostaglandin E_2_ (PGE_2_) and other osteoanabolic mediators—between osteocytes and the extracellular environment, thereby promoting bone formation (Zhao et al., 2023). The importance of this axis was confirmed by genetic ablation: specific deletion of integrin α5 in murine osteoblasts markedly impaired fluid shear stress–induced Cx43 hemichannel activity (Riquelme et al., 2021), establishing integrin α5β1 as a critical upstream regulator of mechanotransductive signaling in bone cells.

Beyond fluid shear stress, other physical cues influence osteogenesis *via* integrin α5β1. Substrate stiffness is critical: matrices with Young’s moduli of 62–68 kPa enhance ERK and Akt phosphorylation, upregulate type I collagen, RUNX2, and osteocalcin (BGLAP), and promote osteogenic differentiation of human MSCs (hMSCs) alongside increased α5β1 expression. Blocking α5 integrin attenuates stiffness-induced osteogenic marker expression, though intriguingly, Akt levels rise—suggesting Akt regulation may involve additional signaling inputs independent of α5β1 (Sun et al., 2018). Moreover, low-intensity pulsed ultrasound (LIPUS) upregulates alkaline phosphatase (ALP) and β-catenin in osteoblasts and osteocytes *via* an α5β1-dependent mechanism, and α5β1 blockade inhibits LIPUS-induced osteogenic protein expression (Watabe et al., 2011).

In summary, integrin α5β1 serves as a critical bridge between cells and the extracellular matrix, playing a central role in skeletal mechanotransduction. Diverse mechanical stimuli—whether fluid shear stress, substrate stiffness, or ultrasound—activate downstream pathways *via* α5β1, including PI3K/AKT and ERK1/2, thereby regulating Cx43 hemichannel function, Runx2 activity, and osteogenic marker expression. Integrity of this mechanosensitive signaling network is essential for normal bone development and remodeling. Elucidating the mechanisms by which integrin α5β1 mediates mechanical cues will not only advance our understanding of bone-metabolic disorders such as osteoporosis but also provide a theoretical foundation for the design of mechanically driven bone-tissue engineering strategies and osteoporosis therapies. Future studies should further investigate the cooperative interactions between α5β1 and other mechanosensors, as well as their regulatory dynamics under different pathological conditions.

## 4 Role of integrin α5β1 in bone pathologic states

Integrin α5β1, as the principal adhesion receptor linking bone cells to the ECM, plays a crucial role in bone physiology (Su et al., 2022). It not only regulates osteoblast differentiation, migration, and adhesion but also mediates signaling during bone remodeling to maintain skeletal homeostasis (Watabe et al., 2011; Su et al., 2018). However, in various bone pathologies, both the function and expression of α5β1 may be altered, resulting in abnormal bone architecture. In conditions such as osteoporosis, osteoarthritis, and bone metastatic disease, α5β1-driven signaling between bone cells can become overactivated or suppressed, thereby exacerbating bone loss (Pantano et al., 2021; Li et al., 2023; Miao et al., 2023). Thus, elucidating the specific mechanisms by which α5β1 operates in these pathological states will enhance our understanding of disease onset and progression and could provide new avenues for targeted bone-disease therapies.

### 4.1 Osteoporosis

Osteoporosis (OP) is a systemic metabolic bone disease characterized by reduced bone mass and the deterioration of bone microarchitecture, leading to increased bone fragility (Lane et al., 2000). Mechanistically, the occurrence of osteoporosis is due to an imbalance between bone formation mediated by OBs and bone resorption mediated by osteoclasts (Prestwood and Raisz, 2002). BMSCs are the primary source of OBs. Maintaining the osteogenic differentiation and proliferation of BMSCs is essential for preserving bone homeostasis. Impaired BMSC function is a decisive factor in the development of osteoporosis (OP) (Qi et al., 2017).

#### 4.1.1 The crucial role of Integrin α5β1 in osteoporosis

Integrin α5β1, as the principal adhesion receptor between bone cells and the extracellular matrix (ECM), plays a decisive role in the onset and progression of osteoporosis. Studies have demonstrated that α5β1 regulates BMSC adhesion, proliferation, migration, and differentiation, thereby directly influencing osteogenesis (Su et al., 2022). In osteoporotic conditions, aberrant expression and function of α5β1 impair BMSC osteogenic differentiation and accelerate bone loss. Gene-knockout and functional-inhibition experiments have confirmed that loss of normal α5β1 markedly reduces bone mineral density and increases fracture risk (Jin et al., 2020; Li et al., 2023). Notably, under reduced mechanical loading or estrogen deficiency, dysfunction of α5β1 signaling is considered a key molecular event driving osteoporosis development (Jin et al., 2020).

Moreover, the β1 subunit—an essential component of the α5β1 heterodimer—is equally critical for maintaining bone integrity. Osteoblast-specific deletion of integrin β1 leads to significant cortical and trabecular bone loss in weight-bearing skeletal regions, underscoring the central role of α5β1 in osteoporotic pathology (Qin et al., 2022).

#### 4.1.2 Molecular mechanisms of Integrin α5β1 in osteoporosis

Integrin α5β1 contributes to osteoporosis pathogenesis *via* multiple signaling cascades, among which the PF4–α5β1–FAK–ERK axis is particularly critical. Platelet factor 4 (PF4), also known as C-X-C motif ligand 4 (CXCL4), belongs to the CXC chemokine subfamily. It is a platelet α-granule protein synthesized by bone marrow megakaryocytes and is associated with OP (Huynh et al., 2019; Li et al., 2023). PF4 can regulate receptor cells through integrins (Lishko et al., 2018). In fibroblasts, PF4 binding to α5β1 promotes ACTA2 expression and drives fibroblast-to-myofibroblast differentiation—a process that is attenuated by integrin inhibitors (cilengitide, echistatin) or α5 knockout (Xiao et al., 2024). In osteoporotic models induced by bilateral ovariectomy (OVX), PF4 was shown to inhibit the α5–FAK–ERK signaling pathway in bone-marrow MSCs (BMSCs), leading to significant reductions in alkaline phosphatase (ALP) activity and impairments in BMSC proliferation and migration. *In vivo*, elevated PF4 expression exacerbated bone loss in OVX mice (Li et al., 2023). These findings delineate a molecular mechanism whereby PF4 disrupts integrin α5β1 downstream signaling to suppress BMSC osteogenic differentiation, thereby accelerating bone mass reduction in osteoporosis.

The mechanisms by which integrin β1 contributes to osteoporosis are likewise of considerable interest. *ITGB1*,the integrin β1 subunit, is the primary subunit that binds to type I collagen in bone and participates in the regulation of skeletal development and function, including stem cell differentiation, articular cartilage structure, and cranial bone ossification (Hughes et al., 1993; Marie et al., 2014; Qin et al., 2020). Zimmerman et al. employed an osteocalcin-Cre system to drive expression of a dominant-negative β1 integrin subunit (β1-DN), thereby partially inhibiting endogenous β1 integrin function *via* competitive interference. This intervention resulted in reduced bone formation rates and decreased bone mass in transgenic (TG) mice, manifested as enlarged cortical porosity and localized thinning of the cranial vault. Notably, Zimmerman et al. observed pronounced sex-dependent differences and a time-dependent recovery phenomenon: at 90 days post-birth, male mice exhibited normalization of parietal bone width, whereas female mice continued to display reduced bone mass (Zimmerman et al., 2000). In contrast, Shekaran et al. used a conditional knockout strategy to delete β1 integrin entirely, systematically examining three distinct developmental stages—mesenchymal condensation, pre-osteoblast, and mature osteoblast—using the same osteocalcin promoter to target mature osteoblasts. They reported only minor alterations in femoral architecture in 10–13-week-old female mice, with bone mineral density, biomechanical properties, and fracture healing capacity remaining essentially normal (Shekaran et al., 2014).

More direct evidence comes from the study by Qin et al., who employed a 10-kb Dmp1-Cre system to delete *ITGB1* specifically in osteocytes. They observed profound cortical and trabecular bone loss in weight-bearing long bones (femur, tibia, and vertebrae), whereas non–weight-bearing cranial bones remained unaffected. Biomechanical testing confirmed that femora from *ITGB1*-deficient mice exhibited significantly reduced bending strength and structural integrity (Qin et al., 2022). Similarly, deletion of other focal adhesion proteins, such as Pinch1/2 and Kindlin-2, produced analogous phenotypes (Wang Y. et al., 2019; Cao et al., 2020),indicating that integrin β1 and its associated adhesion complex proteins form a synergistic signaling ensemble that governs osteocyte mechanosensation and bone remodeling.

#### 4.1.3 Osteoporosis therapeutic strategies targeting Integrin α5β1

Given the central role of integrin α5β1 in osteoporosis pathogenesis, various α5β1-directed therapeutic approaches have been developed. At the genetic level, lentiviral-mediated overexpression of the α5 subunit in human mesenchymal stem cells markedly enhances their osteogenic potential and successfully repairs calvarial defects in murine models (Srouji et al., 2012). Peptidomimetic studies have shown that local administration of the cyclic peptide GA-CRRETAWAC-GA selectively activates integrin α5, upregulates Runx2 and type I collagen expression, and promotes osteoblast differentiation (Fromigué et al., 2012).

Natural compounds have also yielded promising results: Poon et al. demonstrated that icariin binds directly to and activates α5β1, increasing adhesion and integrin expression in bone-marrow stromal cells and effectively preserving osteogenic function under unloading conditions (Poon et al., 2024). Natural compounds have also yielded promising results: Poon et al. demonstrated that icariin binds directly to and activates α5β1, increasing adhesion and integrin expression in bone-marrow stromal cells and effectively preserving osteogenic function under unloading conditions.

In the biomaterials arena, incorporation of α5β1-specific fibronectin fragments into hyaluronic acid (HA) hydrogels significantly enhances MSC adhesion and osteogenic efficiency (Menzel and Farr, 1998; Kisiel et al., 2013). Clinically, bisphosphonates conjugated to HA hydrogels have been engineered as α5β1-targeted drug-delivery systems, achieving sustained release while simultaneously activating α5β1 signaling to promote osteogenic differentiation (Hulsart-Billström et al., 2013; Reid and Billington, 2022). This synergistic approach exemplifies the integration of integrin-targeted therapy with conventional pharmacology.

### 4.2 Osteoarthritis

**FIGURE 3:**
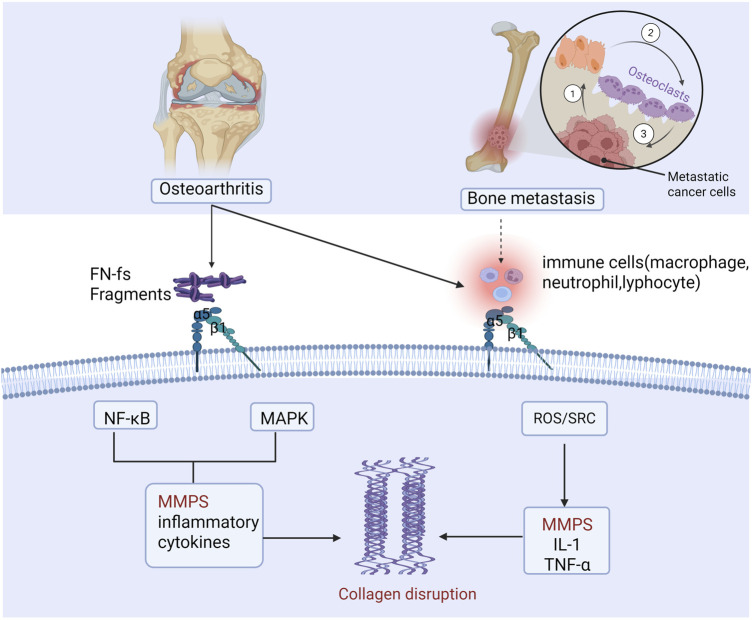
Integrin α5β1 mediates common pathological mechanisms in both osteoarthritis and bone metastasis. In osteoarthritis, cartilage matrix damage generates inflammatory cytokines, matrix metalloproteinases, and fibronectin fragments, which degrade collagen. Fibronectin fragments bind α5β1 and TLRs, activating NF-κB and MAPK signaling to provoke further release of inflammatory mediators and MMPs, driving extensive matrix breakdown. Concurrently, α5β1 engagement on immune cells induces IL-1β, TNF-α, and MMP expression, exacerbating synovial inflammation and cartilage degradation. This same α5β1-driven axis operates in bone metastasis, accelerating tumor-induced bone destruction (figure generated through BioRender.com).

Osteoarthritis (OA) is a prevalent degenerative joint disorder characterized by articular-cartilage degradation, subchondral bone remodeling, and synovial inflammation, leading to chronic pain, joint dysfunction, and activity limitation. As a leading cause of disability worldwide, OA severely compromises patient quality of life (Glyn-Jones et al., 2015; Martel-Pelletier et al., 2016). Chondrocytes, the sole cellular component of articular cartilage, synthesize and secrete intact fibronectin (FN), proteoglycans, and collagens under physiological conditions to maintain matrix homeostasis (Zheng et al., 2021). Integrin α5β1, the principal FN receptor, mediates critical interactions with FN that govern both joint health and OA pathogenesis (Loeser, 2014) (Figure 3). At the molecular level, α5β1 engagement with intact FN *versus* FN fragments activates distinct signaling pathways, resulting in divergent biological outcomes.

#### 4.2.1 Protective interaction between α5β1 and intact fibronectin

Under physiological conditions, chondrocytes secrete intact fibronectin (FN), whose RGD motif binds integrin α5β1 on the cell surface, triggering protective signaling pathways that enhance cartilage-matrix synthesis and suppress excessive matrix degradation, thereby preserving articular-cartilage integrity and function (Qin et al., 2022). Loeser et al. provided direct evidence using an FN-RGD→RGE mutant mouse model (Fn1^RGE/–): under non-loading conditions, both wild-type and Fn1^RGE/– cartilage appeared normal; however, upon high mechanical loading (partial meniscectomy plus forced exercise), Fn1^RGE/– mice exhibited significantly accelerated osteoarthritis progression, with MMP-3 and MMP-13 levels rising by 7.8-fold and 6.4-fold, respectively (Almonte-Becerril et al., 2018). These data demonstrate that disruption of FN–α5β1 binding heightens cartilage vulnerability to mechanical stress and accelerates OA development, confirming the essential protective role of intact FN–α5β1 interactions in maintaining cartilage omeostasis.

#### 4.2.2 Pathogenic interaction between α5β1 and fibronectin fragments

During osteoarthritis progression, extensive cartilage-matrix degradation generates fibronectin (FN) fragments that engage α5β1 *via* mechanisms distinct from intact FN. Miao et al. elucidated this molecular specificity: unlike intact FN, FN fragments binding to α5β1 form endocytic complexes that are trafficked to early endosomes rather than recycling endosomes. These early endosomes co-localize with NADPH oxidase 2 (NOX2) to form specialized “redoxosomes” (Miao et al., 2023). Redoxosomes produce reactive oxygen species (ROS), which activate the redox-sensitive Src kinase, thereby upregulating MMP-13 expression and driving cartilage-matrix degradation (Wood et al., 2016; Miao et al., 2023). Elevated levels of Src and the NOX2 subunit p67^phox in OA patient cartilage further validate the clinical relevance of this pathway (Miao et al., 2023). This work provides the first cellular-biological explanation for how intact FN and its frag.

While Miao et al. demonstrated the crucial role of reactive oxygen species (ROS) in FN-fragment–induced MMP-13 production, the precise redox control of downstream signaling remained unclear. Wood et al. probed this question and uncovered key oxidative-posttranslational-modification mechanisms. They first showed that baseline levels of protein-cysteine S-sulfenylation are markedly elevated in osteoarthritic chondrocytes compared to normal cells, indicating widespread oxidative modifications in OA (Wood et al., 2016). Importantly, treatment of normal chondrocytes with FN fragments induced S-sulfenylation of multiple proteins, notably the tyrosine kinase Src. Using mass spectrometry and immunoblotting, the authors confirmed that FN-fragment exposure not only increased Src S-sulfenylation but also directly enhanced Src kinase activity. Pre-treatment with dimedone—a reagent that specifically traps S-sulfenylated cysteines—or with Src kinase inhibitors effectively blocked FN-fragment–induced MMP-13 production, establishing a causal link in this pathway (Wood et al., 2016). Together, the studies by Miao et al. and Wood et al. delineate a complete signaling cascade: FN-fragment binding to α5β1 → endocytosis into redoxosomes → ROS generation → Src S-sulfenylation → Src activation → MMP-13 upregulation (Popov et al., 2011; Miao et al., 2023).

#### 4.2.3 Multifaceted roles of α5β1 in the joint microenvironment

The pathogenic functions of integrin α5β1 extend beyond chondrocytes to encompass the entire joint microenvironment. α5β1 is also expressed on synovial fibroblasts and immune cells (Lowin and Straub, 2011; Monti et al., 2017). In osteoarthritic synovia, α5β1–fibronectin interactions induce synovial cells to secrete chemokines that recruit and infiltrate macrophages into the synovium and cartilage (Pang et al., 2023). Under α5β1 signaling, these infiltrating macrophages polarize toward a pro-inflammatory M1 phenotype, releasing high levels of IL-1β, TNF-α, and other mediators that further activate synovial and chondrocyte populations, exacerbating synovitis and cartilage degeneration (Attur et al., 2000; Pang et al., 2023). Conditional deletion of integrin α5 in murine joints significantly reduces cartilage damage (Candela et al., 2016), underscoring the central role of α5β1 signaling in OA pathogenesis. Collectively, these studies identify the α5β1 integrin pathway as a critical nexus linking mechanical stress, inflammation, and cartilage degradation across multiple cell types and tissues in osteoarthritis.

#### 4.2.4 Therapeutic potential of targeting α5β1

Given the multifaceted roles of integrin α5β1 in OA pathogenesis, it has emerged as a promising therapeutic target. Early *in vitro* studies demonstrated that RGD peptides (which disrupt α5β1–fibronectin binding) or anti-α5β1 antibodies inhibit FN-fragment–induced chondrocyte MMP production and matrix degradation (Almonte−Becerril et al., 2018). Antisense oligonucleotides (ASOs) targeting *ITGA5* reduce α5β1 expression and MMP-13 generation, thereby slowing OA progression (Homandberg et al., 2002). Moreover, the novel OA disease-modifying candidate LNA043—a derivative of angiopoietin-like protein 3 (ANGPTL3)—has shown in preclinical OA and cartilage-injury models that, by binding α5β1 on MSCs and chondrocytes, it protects and regenerates hyaline cartilage (Gerwin et al., 2022). Curcumin (diferuloylmethane), a bioactive component of turmeric rhizomes with potent anti-catabolic, anti-inflammatory, and antioxidant properties (Ammon and Wahl, 1991), has also been investigated as a potential OA therapy; recent studies indicate that curcumin antagonizes IL-1β–mediated effects and restores integrin β1 expression, thereby enhancing chondrocyte survival (Chin, 2016). Although these approaches remain largely preclinical, targeting integrin α5β1 offers a novel strategy for OA treatment, warranting further investigation into its mechanisms of action and safety profiles.

### 4.3 Bone metastases

Bone metastasis refers to the process by which cancer spreads from the primary site to the bones. This phenomenon is relatively common in various cancers, particularly in breast cancer, prostate cancer, and kidney cancer (Yin et al., 2005; Coleman et al., 2020). During bone metastasis, tumor cells spread to the bones through the blood or lymphatic systems, disrupting OB-mediated bone formation and increasing osteoclast-mediated resorption of mineralized bone (Roodman, 2004). The disruption of this bone homeostasis renders the skeletal architecture fragile and prone to fractures, severely compromising patient quality of life. Bone metastasis is one of the leading causes of mortality in patients with advanced cancer; its pathogenesis is complex and involves the regulation of multiple molecular and signaling pathways.

#### 4.3.1 Role of Integrin α5β1 in bone metastasis of various cancers

Integrin α5β1 exerts distinct biological effects at different stages of tumor bone metastasis. In breast cancer, downregulation of α5 integrin impairs tumor cell adhesion to, migration on, and survival within fibronectin (FN) matrices, thereby reducing the incidence of osteolytic lesions *in vivo*, whereas α5 overexpression enhances bone metastatic colonization (Pantano et al., 2021). Runt-related transcription factor 2 (RUNX2) is a key driver of breast cancer bone metastasis; α5 integrin, as a critical RUNX2 target, augments the chemotactic and adhesive capabilities of breast cancer cells (Li et al., 2016). At the post-transcriptional level, the miR-30 family directly binds the 3′UTR of *ITGA5* to suppress its expression. In triple-negative breast cancer cells with high *ITGA5* expression, knockdown of *ITGA5* or treatment with miR-30 mimics significantly reduces bone-metastatic burden *in vivo*, underscoring the pivotal role of *ITGA5* in breast cancer bone dissemination (Croset et al., 2018). At the epigenetic level, the histone methyltransferase enhancer of zeste homolog 2 (EZH2) upregulates transcription of the β1 integrin gene, thereby activating FAK signaling *via* increased phosphorylation, which in turn modulates TGF-β signaling to promote breast cancer bone metastasis (Zhang et al., 2022).

In other tumor types, integrin α5β1 likewise promotes bone metastasis. In renal cell carcinoma, upregulation of α5 integrin and downstream AKT signaling enhances tumor cell adhesion to extracellular-matrix components, facilitating dissemination to bone (Haber et al., 2015). Prostate cancer cells exhibit high α5β1 expression, and α5 knockout specifically inhibits their migratory and adhesive capabilities during bone colonization (Joshi et al., 2017). Concurrently, RNA epigenetic modification contributes to this process: the methyltransferase METTL3 augments integrin β1 transcription *via* m6A modification, thereby strengthening cancer cell interactions with collagen I and accelerating prostate cancer bone metastasis (Li et al., 2020).

#### 4.3.2 Molecular mechanisms of Integrin α5β1 in bone metastasis

As the principal fibronectin (FN) receptor, integrin α5β1 orchestrates multiple stages of tumor cell dissemination to bone. In the initial phase of bone metastasis, α5β1 specifically binds the abundant FN within the bone marrow stroma, providing essential adhesion sites for circulating tumor cells (Desgrosellier and Cheresh, 2010).

Following adhesion, α5β1 regulates tumor cell migration and invasion within the bone microenvironment (Figure 3). Engagement of α5β1 by FN fragments generated in the marrow induces endocytosis and reactive oxygen species (ROS) production, activating Src/FAK signaling and upregulating matrix metalloproteinases (MMPs) (Mierke et al., 2011; Kiwanuka et al., 2013). Elevated MMP levels degrade the bone matrix, creating space for tumor colonization and liberating embedded growth factors that further support tumor cell survival and proliferation (Pal et al., 2012).

Beyond its direct effects on tumor cells, α5β1 modulates the bone niche by impacting osteoblast and osteoclast activity. Tumor cells exploit α5β1–mediated signaling to secrete a range of cytokines and growth factors—such as TGF-β and RANKL—that disrupt the balance of bone formation and resorption (Pantano et al., 2021; Zhang et al., 2022). This dysregulation establishes a “vicious cycle,” wherein bone destruction and tumor expansion mutually reinforce one another, exacerbating skeletal pathology.

Additionally, RGD-based PET tracers targeting α5β1 have been developed for noninvasive tumor imaging and therapeutic response assessment, showing encouraging results in pancreatic and other cancers and offering a novel tool for monitoring α5β1-directed therapies [136,137].

#### 4.3.3 Therapeutic strategies targeting Integrin α5β1 in bone metastasis

Current Agents and Prospects:Current therapeutic strategies targeting integrin α5β1 in bone metastasis have demonstrated significant potential. Volociximab (M200), the first humanized IgG4 monoclonal antibody against α5β1, binds with high affinity to block the interaction between α5β1 and fibronectin (FN), thereby inhibiting tumor-associated angiogenesis and endothelial-cell proliferation (Ramakrishnan et al., 2006; Pantano et al., 2021). Clinical studies have shown that volociximab, when combined with chemotherapeutic agents such as carboplatin and paclitaxel, exerts synergistic antitumor effects across various solid tumors and holds promise for treating bone metastases (Bell-McGuinn et al., 2011; Almokadem and Belani, 2012; Besse et al., 2013). Similarly, the small-molecule peptide antagonist ATN-161 (Ac-PHSCN-NH_2_), derived from the FN synergy site, competitively inhibits α5β1–FN binding and, in preclinical models of breast and prostate cancer bone metastasis, suppresses MMP-1–mediated tumor invasion, angiogenesis, and osteolysis (Cianfrocca et al., 2006; Khalili et al., 2006; Doñate et al., 2008).

##### 4.3.3.1 Clinical challenges

Despite promising preclinical data, three core challenges hinder the clinical translation of α5β1-targeted therapies. First, tumor resistance limits efficacy: in glioblastoma (GBM), α5β1 suppresses p53 signaling to confer temozolomide resistance (Janouskova et al., 2012), and the β1 subunit activates DNA-repair and anti-apoptotic pathways, endowing cancer cells with chemoresistance—mechanisms likely operative in bone-metastatic cells as well (Eke et al., 2012; Dickreuter et al., 2016). Second, widespread α5β1 expression in normal tissues raises the risk of off-target effects and physiological disruption (Mateo et al., 2014). Notably, while α5β1 activation may promote osteogenesis, excessive stimulation could inadvertently facilitate adhesion and dissemination of dormant tumor cells, creating a therapeutic paradox (Pantano et al., 2021). Finally, safety concerns remain: the α5β1 antibody PF-04605412 triggered severe infusion reactions in clinical trials, highlighting potential immunogenicity and underscoring the need for optimized drug design (Mateo et al., 2014).

### 4.4 Potential roles of other integrins in skeletal disorders

Integrins recognize RGD motifs within the ECM, but different subtypes employ distinct ligand-binding mechanisms. Fibronectin (FN), a major bone matrix protein, contains the RGD sequence within its type III-10 domain, which both α5β1 and αvβ3 can bind (Bharadwaj et al., 2017). However, α5β1 requires cooperation from an adjacent “synergy site” (PHSRN) to enhance affinity, whereas αvβ3 relies primarily on the RGD motif alone to interact with various RGD-containing matrix proteins (Bharadwaj et al., 2017). For example, in osteoblasts, both integrins bind bone sialoprotein, osteopontin, and fibronectin in an RGD-dependent manner. By contrast, α6β4 exhibits a unique structure: its β4 subunit features an exceptionally long cytoplasmic tail (over 1,000 amino acids) comprising multiple FNIII repeats, Calxβ domains, and connecting segments, which mediate hemidesmosome assembly and signal transduction (Stewart and O'Connor, 2015).

Integrins play a diverse role in regulating the complex process of bone metabolism. In addition to integrin α5β1, several other integrins also play key roles in the regulation of bone tissue homeostasis. Notably, as a receptor for type I collagen (Col I), integrin α2β1 serves as a new target for the prevention and treatment of age-related osteoporosis, with a dual role in bone formation (Popov et al., 2011; Stange et al., 2013). In the integrin α2β1 knockout mouse model, the levels of Col I and osteogenic differentiation markers such as RUNX2 and Osterix were significantly elevated (Stange et al., 2013). Additionally, Lumican, a myogenic factor, primarily binds to the integrin α2β1 receptor. By binding to α2β1, Lumican activates the ERK signaling pathway, thereby promoting the differentiation of osteoblasts (OBs). However, integrin α2β1 inhibitors attenuated the stimulation of ERK and ALP activity by Lumican (Lee et al., 2020). The actions of α5β1 and αvβ3 are antagonistic: overactivation of α5β1 inhibits osteoblasts’ ability to form bone matrix, whereas αvβ3 is essential for osteoclast attachment and bone resorption, and its absence impairs osteoclast function (Kokubo et al., 2007). Integrin αvβ3 is an important regulator of osteoclast differentiation and resorption. Blocking αvβ3 can affect signaling pathways within osteoclasts (including c-Src, Pyk2, *etc.*) and may serve as a potential intervention target for osteoporosis and tumor bone metastasis (Nakamura et al., 2007; Lin et al., 2017). A highly selective αvβ3 integrin antagonist (HSA-ARLDDL, derived from snake venom protein modification) can effectively inhibit RANKL-induced osteoclastogenesis and reduce bone loss in ovariectomized mice, without interfering with osteoblast differentiation (Lin et al., 2017). In bone metastatic tumors such as breast cancer and prostate cancer, the overexpression of αvβ3 promotes tumor colonization in the bone environment and induces osteolytic destruction (McCabe et al., 2007; Zhao et al., 2007). The αvβ3/αvβ5 dual inhibitor Cilengitide has shown some efficacy in a breast cancer bone metastasis model: although *in vivo* administration did not completely prevent bone metastasis, it significantly reduced the volume of osteolytic lesions and shrank the size of bone metastatic tumors (Bretschi et al., 2011). Overall, existing evidence supports integrin αvβ3 as a promising target for the treatment of osteoporosis and bone metastasis, but how to fully and safely utilize this target remains a research hotspot. Integrins α5β1 and α4β1 on synovial cells also crosstalk with each other, jointly regulating the production of pro-inflammatory mediators and MMPs (Jin et al., 2021). Additionally, integrin αvβ3 has recently been found to be involved in the regulation of arthritis inflammation and cartilage degeneration: studies indicate that the dysregulation of the αvβ3/CD47 signaling axis in OA exacerbates joint inflammation and cartilage destruction (Wang Q. et al., 2019). In addition, integrin α10β1 is the primary type II collagen-binding receptor on chondrocytes and has been shown to enhance cartilage formation potential. Studies indicate that intra-articular injection of MSCs with high integrin α10 expression can alleviate post-traumatic osteoarthritis damage (Delco et al., 2020). This also suggests that targeting integrin signaling could become a new approach for intervening in OA.

Overall, each integrin serves as both an entry point for basic research and a potential therapeutic breakthrough. The role of integrin α5β1 alone in bone disease treatment may be minimal and singular. Therefore, in the future, it is likely that targeting multiple key integrin pathways in combination will intervene in the bone microenvironment, thereby preventing and treating the occurrence and progression of bone diseases.

## 5 Summary and outlook

### 5.1 Summary of findings

Integrin α5β1, as a key receptor linking cells to the extracellular matrix, plays a central role in both the maintenance of bone-tissue homeostasis and the pathogenesis of skeletal disorders. Under physiological conditions, α5β1 mediates the differentiation of mesenchymal stem cells into osteoblasts, regulates osteoblast adhesion and migration, and promotes matrix synthesis and mineralization, thereby orchestrating bone development and remodeling. Notably, as a mechanosensor, α5β1 converts physical forces into biochemical signals that drive adaptive bone formation in response to mechanical stimuli.

In pathological states, dysregulation of α5β1 function is closely associated with multiple bone diseases. In osteoporosis, downregulation of α5β1 impairs osteogenic activity; in osteoarthritis, fibronectin fragments activate chondrocyte α5β1 to induce matrix degradation; and in bone metastasis, tumor-cell overexpression of α5β1 facilitates colonization of the bone microenvironment. These insights not only deepen our understanding of skeletal disease mechanisms but also underscore the therapeutic potential of targeting integrin α5β1.

### 5.2 Overview of the Integrin α5β1 signaling network

The integrin α5β1 signaling network exhibits a three-tiered architecture: an upstream regulatory layer, a core signaling cascade, and a downstream effector layer. The upstream layer fine-tunes α5β1 expression *via* noncoding RNAs, epigenetic modifiers, and transcription factors; the core signaling tier transduces ligand engagement into intracellular messages through FAK/Src, ERK/MAPK, and PI3K/Akt pathways; and the downstream effector stratum governs critical osteoblastic functions such as differentiation, adhesion, and matrix remodeling. Table 1 summarizes the key regulatory components at each level—including expression modulators, ligands/regulators, and clinical intervention agents—providing a comprehensive framework for understanding the multilayered control of integrin α5β1 in bone tissue (Table 1).

**TABLE 1:** Integrin α5β1 roles and disease impacts by cell type.

Cell type	Role of Integrin α5β1	Impact in disease	References
MSCs (Mesenchymal Stem Cells)	Promotes differentiation into osteoblasts, enhancing osteogenesis and repair	Facilitates MSC differentiation and tissue regeneration	Ono et al. (2011), Di Maggio et al. (2017)
Osteoblasts	Regulates bone formation, participates in matrix deposition and mineralization	Overactivation can exacerbate bone loss and stimulate osteoclast activity	Oh et al. (2017), Xiao et al. (2024)
Chondrocytes	Modulates cartilage-matrix synthesis and degradation, and promotes inflammatory responses	In OA, drives cartilage degradation and exacerbates joint inflammation	Wood et al. (2016), Qin et al. (2022)
Cancer Cells	Enhances adhesion, migration, and invasion, contributing to metastasis	Promotes tumor cell invasiveness and bone metastasis	Li et al. (2016), Pantano et al. (2021)

### 5.3 Knowledge gaps and future outlook

Although integrin α5β1 has been studied in bone metabolism and related diseases, several key scientific questions remain to be addressed: (1) In pathological conditions such as osteoporosis, how does α5β1 simultaneously affect the balance between osteogenesis and osteoclastogenesis, and how can precise delivery systems be developed to avoid adverse effects on bone formation? (2) In osteoarthritis, how can the α5β1 pathway be selectively regulated to suppress harmful signals while preserving cartilage-repair functions? (3) Can high α5β1 expression serve as a biomarker for patient stratification to predict responses to integrin-targeted therapies? (4) How do transcription-factor networks and epigenetic modifications precisely regulate α5β1 expression in different bone cell types, and how are these mechanisms altered in disease states?

To address these gaps, future precision interventions may be developed at the three levels outlined in Table 2: (1) Upstream regulatory layer: Fine-tune α5β1 expression by modulating miRNAs (e.g., miR-30) or epigenetic modifiers (e.g., EZH2, METTL3); (2) Core signaling layer: Develop selective modulators targeting α5β1 downstream pathways (e.g., FAK/ERK) to promote osteogenesis or inhibit bone resorption; (3) Downstream effector layer: Design disease-specific interventions, such as blocking FN-fragment binding to α5β1 in OA or developing partial agonists to selectively enhance osteogenesis in osteoporosis.

**TABLE 2:** Key regulators of Integrin α5β1 and their functions.

Regulatory tier	Regulator	Type	Mechanism of Action	References
**Upstream Control**	circRNA-VGLL3	circRNA	Upregulates *ITGA5 via* a miRNA-sponging mechanism, enhancing osteogenic potential	Notni et al. (2016)
	miR-30	miRNA	Suppresses integrin α5 expression, reducing bone-metastatic invasiveness of breast cancer cells	Coleman et al. (2020)
	EZH2	Histone methyltransferase	Enhances β1 integrin gene transcription, promoting breast cancer bone metastasis	Yin et al. (2005)
	METTL3	m^6A RNA methyltransferase	Promotes integrin β1 expression *via* m^6A RNA modification, accelerating prostate cancer bone metastasis	Eke et al. (2012)
Ligands/Regulators	CCN3	ECM protein	Activates α5β1 signaling to upregulate Runx2 and osterix expression, driving osteogenesis	Urist (1965)
	WISP-1	ECM protein	Enhances BMP-2 binding to BMSCs *via* α5β1, promoting bone formation	Loeser (2014)
	BMP-2	Growth factor	Synergizes with α5β1 to amplify osteogenic signaling, promoting differentiation	Cao et al. (2020)
	Runx2	Transcription factor	Activates *ITGA5* transcription, facilitating osteogenic differentiation	Kiwanuka et al. (2013), Shao et al. (2019)
	NF-κB	Transcription factor	Activates *ITGB1* transcription, enhancing inflammation and cell adhesion	Kisiel et al. (2013)
Intervention Agents	Icariin	Natural product	Activates α5β1 to enhance bone formation and cell adhesion	Shekaran et al. (2014)
	GA-CRRETAWAC-GA	Synthetic peptide	Activates α5β1 to promote expression of osteogenic genes	Zimmerman et al. (2000)
	Volociximab (M200)	Monoclonal antibody	Blocks α5β1–fibronectin binding, inhibiting angiogenesis and metastasis	Haber et al. (2015), Joshi et al. (2017)
	ATN-161	Integrin-inhibitory peptide	Disrupts α5β1–fibronectin interaction, suppressing tumor invasion and bone metastasis	Desgrosellier and Cheresh (2010), Mierke et al. (2011)

Multidisciplinary collaboration—integrating biomechanics, pharmacology, and materials science—will be essential to translate α5β1-targeted strategies into clinical practice. Combination therapies (e.g., α5β1 antagonists with anti-bone-metastasis agents) or novel delivery systems hold the promise of synergistic effects, providing more effective and safer treatment options for osteoporosis, osteoarthritis, and bone metastasis.
